# A Delivery Model for Home Fortification of Complementary Foods with Micronutrient Powders: Innovation in the Context of Vietnamese Health System Strengthening

**DOI:** 10.3390/nu8050259

**Published:** 2016-04-29

**Authors:** Marie Nguyen, Alia Poonawala, Magali Leyvraz, Jacques Berger, Dominic Schofield, Tran Thuy Nga, Tran Khan Van, Do Thi Bao Hoa, Frank Tammo Wieringa

**Affiliations:** 1 Global Alliance for Improved Nutrition (GAIN), Geneva 1211, Switzerland; n.guyen_marie@yahoo.fr (M.N.); apoonawala@gainhealth.org (A.P.); magali.leyvraz@gmail.com (M.L.); dschofield@gainhealth.org (D.S.); 2 Institute of Research for Development (IRD), UMR 204 Nutripass IRD-UM-SupAgro, Montpellier cedex 34394, France; jacques.berger@ird.fr; 3 National Institute of Nutrition (NIN), Hanoi, Vietnam; thuynga1997@gmail.com (T.T.N.); khanhvan206@yahoo.com (T.K.V.); dothibaohoa@gmail.com (D.T.B.H.)

**Keywords:** IYCF, delivery model, sustainability, micronutrient powders, health system, complementary feeding, Vietnam

## Abstract

Adding micronutrient powders (MNP) to complementary foods at the point of preparation (home fortification) can improve micronutrient status of young children. Ensuring sustained access to MNPs at scale, however, remains challenging in many countries. The Global Alliance for Improved Nutrition (GAIN) partnered with the National Institute of Nutrition (NIN) in Vietnam to pioneer the distribution of a locally-produced MNP, provided for sale through the public health system with counseling on optimal infant and young child feeding practices by trained health workers. Different packaging options were available to adapt to caregivers’ disposable income. During the six-month pilot, 1.5 million sachets were sold through 337 health centers across four provinces, targeting children 6–59 months of age. Sales were routinely monitored, and a cross-sectional survey in 32 communes for caregivers (*n* = 962) and health staff (*n* = 120) assessed MNP coverage and compliance, five months after the start of distribution. A total of 404 caregivers among the 962 caregivers surveyed (*i.e.*, 42%) had visited the health center in the past year. Among them, 290 caregivers had heard about the product and a total of 217caregivers had given the MNP to their child at least once, representing a conversion rate from product awareness to product trial of 74.8%. The effective coverage (*i.e.*, consumption of ≥3 sachets/child/week) was 11.5% among the total surveyed caregivers and reached 27.3% amongst caregivers who visited health centers in the previous month. The MNP purchase trends showed that the number of sachets bought by caregivers was positively correlated with the wealth index. The pilot showed that providing MNPs for sale in packs of various quantities, combined with infant and young child feeding (IYCF) counseling at the health center, is effective for groups accessing the health system.

## 1. Introduction

### 1.1. Background

Following important market-oriented reforms in the late 1980s, Vietnam experienced rapid economic growth and, since 2010, the country has been a lower-middle-income country. Poverty rates have fallen from 58% in the early 1990s [1] to 11% in 2012 [2]. In parallel with socioeconomic development, the population of Vietnam has seen significant improvements in nutritional status, especially among children under five years of age. Malnutrition rates have fallen considerably, with a decrease in the prevalence of underweight from 45% in 1990 to 27% in 2004 [3] and a halving of the prevalence of stunting in only 20 years (from 60% in 1985 to 30% in 2005 [4]). In addition, nutrient intakes have increased significantly [5], and anemia prevalence decreased from 45.5% in 1995 [6] to 29.2% in 2009 [7].

Despite these encouraging developments, research conducted in 2010 indicated that the prevalence of iron deficiency, vitamin A deficiency, and vitamin D deficiency among children under five years of age remained high (13%, 29%, and 57%, respectively) [8]. Furthermore, zinc deficiency (low plasma zinc concentrations), found among 80% of children aged 6–75 months in Vietnam, is a severe public health problem. Other deficiencies, such as folate and vitamin B12, were found especially high among children aged 6–17 months [8]. In this regard, micronutrient deficiencies remain a public health issue in Vietnam, contributing to the multiple burdens of malnutrition present in the country; affecting not only undernourished populations, but those who are overweight, as well [9].

Nutrition is considered a national priority and the Government of Vietnam has been developing national plans to improve food security and nutrition since the early 1990s. The most recent National Nutrition Strategy (NNS) (2011–2020) endorsed by the Prime Minister [10] includes a micronutrient deficiency control program that aims to gradually increase the level of vitamins and minerals consumed and absorbed to adequately meet daily needs. Interventions to address vitamin A, iron, and iodine deficiencies among high-risk populations have been prioritized [10]. A national biannual vitamin A supplementation program for all children 6–36 months and for children between 36 and 60 months old living in areas with a high prevalence of vitamin A deficiency has been implemented since 2007. Other interventions include periodic deworming and, in selected areas, iron/folate supplementation for pregnant women and women of reproductive age and zinc supplementation for children with diarrhea [11]. The country is also working on promoting and recommending the use of iodized salt and micronutrient-fortified food (such as flour, fish sauce, condiments, and oil) to increase micronutrient intake at scale [12].

However, the resources invested to address malnutrition have not yet met the needs of the population and have not allowed the NNS to achieve comprehensive and synchronized nationwide implementation. The National Institute of Nutrition (NIN), an agency of the country’s Ministry of Health (MOH) that focuses on nutrition policy and program implementation, has also recognized that nutrition programming through the public health sector remains weak due to poor training of staff and the lack of prioritization of micronutrient deficiency prevention in the national and provincial budgets [10].

### 1.2. Home Fortification Pilot Project

To complement the NNS interventions already addressing micronutrient deficiencies, NIN, and the Global Alliance for Improved Nutrition (GAIN) launched a project of home fortification of complementary foods with micronutrient powders (MNPs) in 2013 with the ultimate goal of strengthening the current public health system. The use of home fortification of complementary foods with MNPs is a proven, effective solution to help decrease micronutrient deficiencies and is endorsed by UNICEF and the World Health Organization (WHO) [13,14]. Home fortification of complementary foods with MNPs has proven to reduce anemia by 31% and iron deficiency by 51% in children 6–24 months [15]. As the research conducted in 2010 showed that some micronutrient deficiencies, such as zinc and vitamin A, were still high among children above two years old, and as the target group of MNP should be those who are at risk of having an inadequate intake of micronutrients; it was decided to target children from 6 to 59 months with the MNP to be able to address these deficiencies [8,14]. The project aimed to increase access of caregivers of children under five years old to essential vitamins and minerals, in the context of the promotion of infant and young child feeding (IYCF) practices by trained health workers and nutrition counsellors. Based on a thorough business plan and scoping exercise, this was the first attempt by the government to design a sustainable health system approach for improved complementary feeding, whereby locally-manufactured MNPs were sold through the local health centers in four pilot provinces. The intent of the model was to create a sustainable means of distributing adequate quantities of MNP to the caregivers of Vietnamese children. The initiative relied on national data provided by NIN, confirming that more than 90% of caregivers visited the health center at least once in the first two years of the child’s life (mainly for immunization purposes [16]). Then a landscape assessment and multiple meetings at national and provincial level with the NIN, key stakeholders, caregivers and commune level health staff provided further input into the business plan of the distribution model. The assumption was that with proper counseling from health workers and an affordably priced product for caregivers, the model would be sustained beyond the duration of the pilot.

#### 1.2.1. Product Development

The MNP product, branded Bibomix, was produced by NIN’s Food and Technology Center (NINFood), NIN’s research and development arm. The product contained 15 essential vitamins and minerals, packaged in three different sizes: a single 1 g sachet, a packet of 10 sachets, and a box of 60 sachets. NINFood formulated and labeled Bibomix based on Home Fortification Technical Advisory Group (HF-TAG) guidelines [17] and detailed in Table 1.

Formative research on the Bibomix packaging, as well as consumer testing (by focus group discussions with caregivers), were conducted to improve the branding and packaging, increase the product’s overall appeal, and make it relevant and desirable to caregivers in the context of IYCF counselling through the public health system.

Bibomix was sold at an end-consumer price of 2000 VND (about US$0.09) per single sachet, 20,000 VND (about US$0.90) for a packet of 10 sachets, and 120,000 VND (about US$5.40) for a box of 60 sachets. The price and the packaging size, pre-tested with caregivers, allowed flexibility for the caregivers according to their available income on the day of the visit to the health center, and also included a margin at every level of the distribution network to cover product, communications, and logistics costs, thereby making the model financially sustainable.

#### 1.2.2. Health System Strengthening and Behavior Change Interventions

While a multichannel communication plan was designed, the initial focus was to ensure that the agents of product delivery, *i.e.*, the health workers, were effectively trained on home fortification of complementary foods with MNPs and on optimal IYCF and water, sanitation, and hygiene (WASH) practices. It was critical that all health workers were equipped with sufficient knowledge to provide appropriate and comprehensive care and nutrition counseling to the caregivers, as well as to recommend the correct use of Bibomix. From June to early August 2014, NIN conducted intensive health staff trainings for almost 1000 health workers; NIN coordinated the implementation in the provinces, aligned and integrated with existing nutrition counseling training sessions, to allow for continuity in the training curriculum. The counseling services were free of charge for the caregivers.

Finally, visibility materials (e.g., posters, educational fans, and product displays) were developed to drive awareness of Bibomix, support the counseling, and educate caregivers on correct use of the product. Before being finalized and printed/produced, all materials were pretested through focus group discussions to assess the understanding of content, the relevance of the designs, and the level of emotional engagement and sensory reactions from caregivers.

#### 1.2.3. Distribution

The local commune health centers (CHCs) were chosen as the main distribution channels, primarily because they are the main access points for public health services and because their local health staff are trusted and respected by caregivers. Therefore, CHCs could become places where caregivers would receive both counseling services and MNP from local health staff.

**Figure 1:**
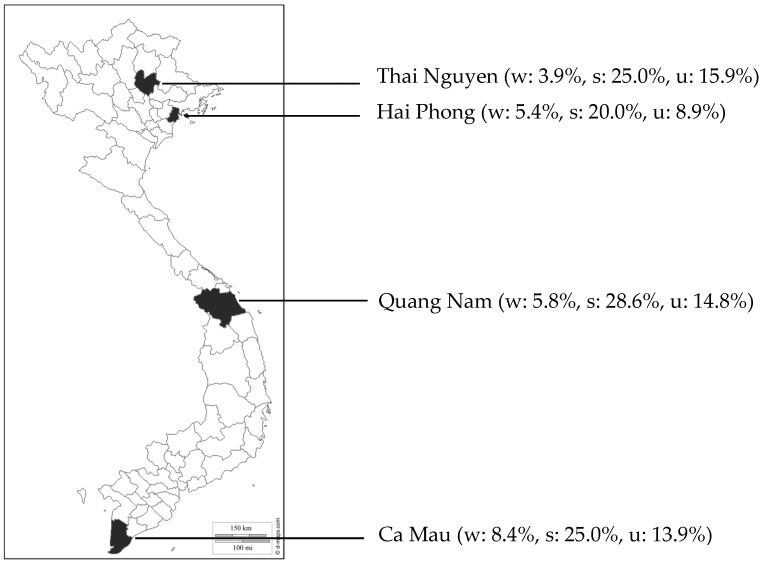
Provinces selected in the pilot and their malnutrition prevalence in 2013. With w: wasting rate, s: stunting rate, and u: underweight rate.

In consultation with NIN, four provinces were chosen from across the country based on the malnutrition rates among children under five years of age. The provinces selected were Hai Phong, Thai Nguyen in the North, Quang Nam in the center and Ca Mau in the South. Their geographical locations as well as respective malnutrition prevalence at baseline (*i.e.*, in 2013) are detailed in the Figure 1 [18]. The provincial health department of each province proposed a list of districts and communes after local consultations determining their willingness to join the pilot project and on the accessibility of their health centers. To this end, supplies of Bibomix, along with educational and visibility materials, were distributed to 337 local health centers from July 2014 to December 2014. The distribution was entirely integrated within the health system, leveraging existing meetings and platforms to supply the health centers, going from province to district, district to commune, and finally commune to local health center.

#### 1.2.4. Objectives

This article presents the pilot’s design, implementation, coverage results, and MNP use and compliance by caregivers. The article then provides recommendations on how the results from this pilot could help inform the strategy on home fortification of complementary foods with MNPs for micronutrient deficiency prevention, and how this model could be scaled up in Vietnam.

## 2. Methods

The recommendations in this article are based on data collected from three sources: NIN monitoring data (e.g., sales and distribution indicators); a qualitative survey with health workers; and a quantitative coverage survey with caregivers. Table 2 provides a summary of the project design, sampling size, and implementation time frame.

### 2.1. Coverage Survey Details, Sampling Method, and Sampling Size Calculation

The coverage survey consisted of structured interviews for the caregivers that were adapted from GAIN’s standard Fortification Assessment Coverage Tool (FACT) and composed of different validated modules.

A stratified multistage cluster sampling with probability proportional to size (PPS) was used to select caregivers. At the first stage, two districts were selected per province by simple random sampling (SRS). In the second stage, four communes per selected district were selected. Each commune was considered as one primary sampling unit. A total of 32 communes were selected. In the final stage, children from 6 to 60 months old were selected by SRS in each commune from the list of the children under 5 years old living in the 32 selected communes.

The primary survey respondents were caregivers of the selected children. To get a precision of 5.0% with an expected design effect of 2.0 and assuming the effective coverage would be around 8%, the minimum sample size was 230 children per province, *i.e.*, 920 children in total and 29 children per commune. To allow a 15% drop-out or non-response rate (caregivers not coming to the CHC or refusing to respond), 35 children per commune were selected. For the health workers, all the health staff at the selected provinces, districts, and communes who were trained were recruited to participate in the in-depth health worker interviews.

### 2.2. Coverage Survey Instruments and Indicator Definition

The coverage terminology is defined as follows:

- Message coverage: percentage of caregivers who have ever heard of or seen Bibomix.
- Contact coverage: percentage of children under five years who have ever tried Bibomix.
- Partial coverage: percentage of children under five years who consumed at least one sachet of Bibomix over the past week.
- Effective coverage: percentage of children under five who consumed at least three sachets of Bibomix over the past week.

The three sachets per week consumption frequency was calculated assuming 60 sachets over six months as recommended by WHO (two months consuming the MNP daily and four months without MNP, [13]). These guidelines, also recommended by the Home Fortification Technical Advisory Group Programmatic Brief were adapted to be more practical from a program implementation perspective by allowing more flexibility of purchase and use by consumers [19]. The 60 sachets over six months represents an average consumption of 10 sachets per month, and 2.5 sachets per week, which was round up to three sachets per week for ease of communication by health workers, and to be sure that the recommended levels of consumption would lead to nutritional impact [20].

Caregivers were asked to recall the MNP consumption over the past week. A one-week recall was chosen to avoid a recall for a longer period, which could result in significant recall bias and other related errors.

The MNP compliant use was assessed among the caregivers. Compliance was defined if all of the following conditions were met:

- The caregiver gave one full sachet of MNP on the most recent day.
- The MNP sachet was mixed into one meal.
- The MNP sachet was added separately for the older infant or young child with mashed family food or was added with semi-solid food prepared for the infant/child.
- The MNP sachet was added just before serving the child a meal.

In addition, data were collected on the household assets and amenities, to allow us to construct a wealth index. A principal component analysis (PCA) was run, and to construct the wealth index, the principal component (first factor) was taken to represent the household’s wealth. For further analysis, the sampling was weighed by household size, and the wealth index was divided into quintiles and ranked into five categories, from the poorest to poor, medium, wealthy, and the wealthiest. The wealth index quintile was applied to each survey respondent who was classified into one of the five categories.

### 2.3. Ethical Considerations for the Coverage Survey Implementation

The survey protocol was approved by the NIN scientific and ethical committee, and signed by Associate Professor Le Danh Tuyen, Director of the NIN, on 28 November 2014 (Ethical Clearance Approval number: 1615/QD-VDD). In addition, the provincial local authorities, as well as the directors of all selected commune health centers, granted permission for the survey. Signed informed consent was obtained from all participating caregivers and health workers. Within the survey database, the respondents were anonymized and identified through unique identifier numbers.

### 2.4. Coverage Survey Data Collection, Entry, and Analysis

Data collection forms and questionnaires were pretested and translated into Vietnamese. Surveyors were trained for one day on the data collection forms/questionnaires. For both caregivers and health workers, the surveyors administered the questionnaire only after having obtained signed informed consent from the respondent. The survey took place in four provinces (Thai Nguyen, Hai Phong, Quang Nam, and Ca Mau) in eight districts and 32 communes.

Data entry was done in Epidata v.3.1 (The EpiData Association, Odense, Denmark). To reduce entry errors, data entry screens were programmed to accept only predetermined codes or values within a given range for each variable. Data analyses were conducted with SPSS software (SPSS 19.0 for Windows, Chicago, IL, USA). Summary enrollment characteristics of the children and caregivers were calculated as means ± standard deviation (SD) for continuous measures. Differences in prevalence were tested with chi-square statistics and associations between variables were tested with Pearson correlation. A *p*-value of < 0.05 was considered significant.

## 3. Results

### 3.1. Routine Monitoring Results

MNPs were delivered for sale by health center staff for approximately six months to a total of 337 health centers, including four provincial health centers (one in each province), 20 district health centers, and 313 CHCs. In Hai Phong, 7 district health centers and 128 CHCs participated in the pilot; in Thai Nguyen, three district health centers and 37 CHCs participated; in Quang Nam, six district health centers and 94 CHCs participated; and in Ca Mau, four district health centers and 54 CHCs participated.

More than 1000 training manuals, 1000 booklets, and 340 counseling cards were developed and produced for the health workers to support their training and counseling. In addition, 340 product displays, 300 posters to drive awareness of MNP, 300 posters to educate on correct use of MNP, and 25,000 educational fans (on home fortification of complementary foods with MNPs) were produced and delivered to the selected commune health centers, together with the guidelines on how to use or distribute these materials.

MNP sales in the four provinces during the project timeline varied by province, with a total of 1,000,500 sachets sold through the pilot and an additional 497,940 sachets sold to other national nutrition programs during the pilot intervention. Thus, the target of 1.5 million sachets in the initial business plan was reached.

The main purchaser of the MNP was the primary caregiver, *i.e.*, the mother (99.4%). Caregivers from the northern provinces, Hai Phong and Thai Nguyen, bought Bibomix in full 60-sachet boxes (52% and 71% of caregivers, respectively), while 74% of caregivers from the center province (Quang Nam) bought the product mainly in 10-sachet packets. In the southern province (Ca Mau), 49% of caregivers bought single sachets and 43% bought packets of 10 sachets.

### 3.2. Health Worker Survey (Monitoring and Coverage Survey) Results

A total of 991 health workers were trained on IYCF, WASH, and home fortification of complementary foods with MNPs (all health workers who were contacted attended the training). A large majority of the health workers (80.8%) who were trained reported having counseled caregivers at their last visit to the health center. The mean duration of one counseling session was 13.9 ± 7.4 min (no significant differences among the provinces).

**Figure 2:**
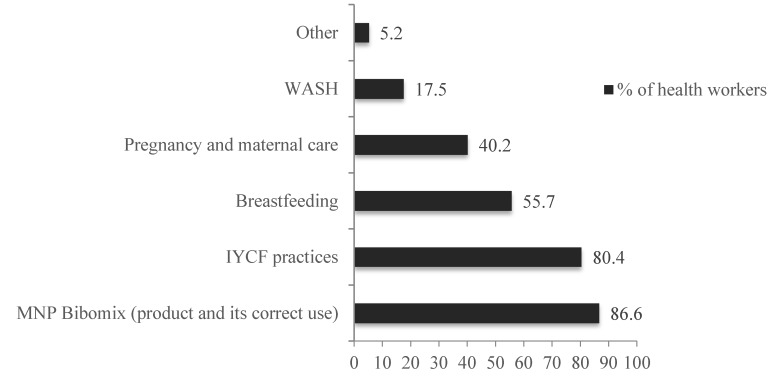
Counseling topics that were provided to the caregivers, as reported by the health workers.

Health workers were questioned about the counseling topics they provided to the caregivers and had the possibility to give multiple responses. The main counselling topics provided to caregivers were on IYCF, MNP, and breastfeeding, as detailed in Figure 2, demonstrating that Bibomix was well integrated with the promotion of optimal IYCF practices.

The majority of the health workers reported using BCI materials as a support for their counseling, with the majority of them using the training manual and the counseling cards. Specifically, 72.8% of the health staff reported using counseling cards, 69.9% used the training manual, 65.2% used the educational fan, and 56.5% used the educational poster; evidence that most materials were used in compliance with the guidelines.

Notably, only 24 health staff (20%) did not encounter any difficulties promoting the MNP to caregivers. For the remaining majority, a variety of barriers to sales, thereby impeding effective coverage, were reported: 11.7% of health workers reported that product usage, *i.e.*, mixing Bibomix into semi-solid foods, was particularly difficult for feeding children over two years old, as these children were not eating semi-solid food anymore; 11.5% of health workers stated that the price of Bibomix was found to be too expensive for caregivers (no significant differences were found between provinces); the lack of rapid and visible benefit/impact on the child’s health after using Bibomix was also raised as a barrier to sales by five health staff. These health workers reported counseling caregivers that improvement in nutritional status with MNP consumption has been proven, but that it is a long-term process and that the product must be used for at least six months to be effective. Eight health workers reported that they encountered caregivers who stopped using Bibomix because of some side effects they attributed to the MNPs use, such as vomiting and diarrhea. The link between MNP consumption and side effects was not proven, and the health staff did their very best to address the problem, providing the required referrals and reassuring the caregivers. Finally, 8.3% of health staff reported that it was difficult to sell the MNPs to caregivers because Bibomix had a lot of competitors and there were many other similar products to Bibomix that were better known.

During field trips to the provinces, the health workers also mentioned that the current guideline on the MNP ordering cycle and supply was quite difficult for them to implement. Indeed, health staff did not forecast the MNP orders “optimally”, as for instance they ordered too much product during their 1st order to NIN which led to budget issues regarding paying for MNP supplies, as they did not sell all the boxes ordered the first month (while the policy was to pay for the MNP based on its order, not on its sales). Although it happened only rarely, according to some health staff, transportation challenges also lead to a shortage of Bibomix in three out of 337 health centers (one in Hai Phong, and two in Quang Nam provinces). None of the supply shortage at health centers was due to lack of production capacity at the plant, but rather to underestimations during forecasting and payment delays.

### 3.3. MNP Coverage and Compliance Use among Mothers of Children under Five Years

The coverage survey at the end of the pilot study included a random sample of 962 surveyed caregivers with children from 6 to 60 months meeting the inclusion criteria. Table 3 presents the characteristics of the surveyed children and their caregivers.

The MNP coverage survey during the pilot implementation showed that 30.1% of the surveyed caregivers had ever heard about Bibomix, 22.6% of them had ever given the product to their child, 21.7% of them had given some of it to their child (at least one sachet per week), and 11.5% used the product effectively (*i.e.*, at least three sachets per week for the child).

**Figure 3:**
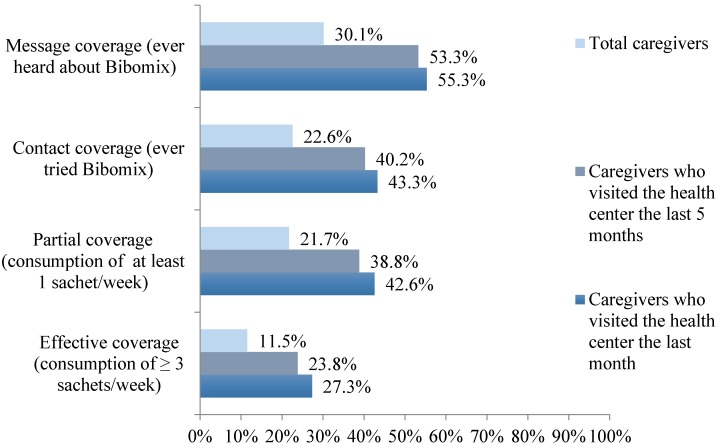
Coverage of the pilot in the four provinces by visit to the health centers.

MNP message coverage was found higher among the caregivers who had visited the health center the month prior to the coverage survey (*p* = 0.003) as illustrated in the Figure 3, which shows that the health center as a delivery channel for the intervention was a key success factor to guarantee effective MNP coverage.

The caregivers’ access to the health center differed greatly by province, with Ca Mau having the lowest rate of caregivers visiting the health center within the last five months while Hai Phong had the highest, 18.2% and 68.8%, respectively (*p* < 0.001).

MNP coverage differed significantly by province, with Hai Phong having the highest coverage rates and Ca Mau the lowest (*p* < 0.05). Message coverage reached 44.2% of caregivers in Hai Phong, 32.6% in Quang Nam, 27.5% in Thai Nguyen, and 17.4% in Ca Mau. The percentage of caregivers who ever gave the MNP to their child (*i.e.*, contact coverage) was 28.6% in Hai Phong, 26.9% in Quang Nam, 20.8% in Thai Nguyen, and 14.6% in Ca Mau. In addition, 26.8% of caregivers in Hai Phong, 26.9% in Quang Nam, 19.1% in Thai Nguyen, and 14.6% in Ca Mau had given at least one sachet of MNP per week to their child. Finally, the effective use of the MNP (*i.e.*, at least three sachets per week for the child) was found among 17.7% of caregivers in Hai Phong, 12.8% of caregivers in Quang Nam, 11% in Thai Nguyen and 5.1% in Ca Mau. In Ca Mau province, while fewer than 20% of the caregivers had heard about the MNP and the effective coverage was only 5.1%, the conversion rate from product awareness to product trial was the highest, with 84.0% of the caregivers who had heard about the MNP Bibomix trying it. For the remaining provinces, the conversion rate was 65.0% in Hai Phong, 72.0% in Thai Nguyen, and 82.0% in Quang Nam (*p* = 0.02).

**Figure 4:**
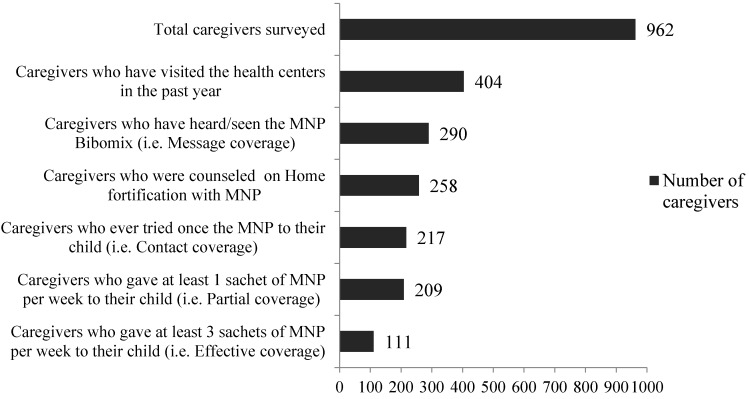
Caregivers visit and counseling at the health centers and MNP coverage.

The results of the caregivers visit and counseling at the health centers, as well as the MNP coverage, are summarized in the Figure 4.

In terms of compliant use, caregivers of children under five years of age using the MNP reported adding it just before the meal was served (86%), adding it to semi-solid food (85% total, and 82% among caregivers with children above two years old), mixing the MNP into one single meal (80%), and giving one full sachet per day (84%). When combining all optimal use behaviors, results show that the majority of the caregivers surveyed (59.0%) were compliant with all the recommended product use behaviors.

Among the 290 caregivers who had heard of MNPs, 74.8% of them had tried it for their child. The most frequently mentioned reasons why the 73 caregivers who had heard of Bibomix did not try it were: (1) “Don’t see the need for Bibomix” (25%); (2) No specific reason given (18%); (3) “I am using another product” (12%); and (4) “I haven’t seen Bibomix at the health center” (11%). In addition, among the 209 caregivers partially covered (*i.e.*, who use the MNP for their child at least once per week), only 16 caregivers reported that they stopped buying the MNP, mainly due to child refusal (56.2%), lack of perceived need (25%), price (6.2%), “do not see impact on child’s health” (6.2%), and side effects (6.2%).

**Figure 5:**
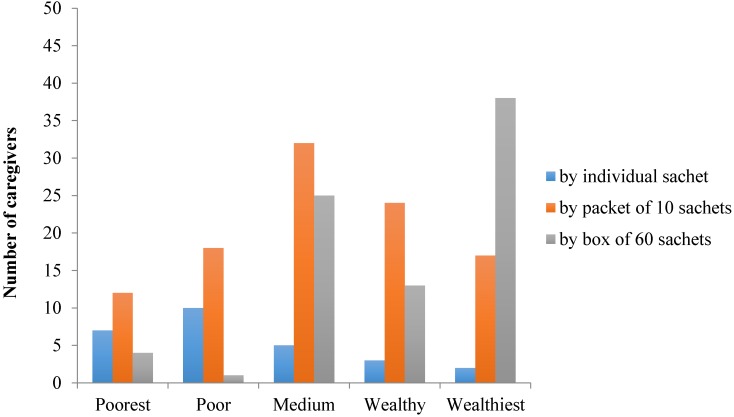
MNP purchase trends as reported by the caregivers, by wealth index quintiles.

Households’ assets and amenities were analyzed in order to apply a wealth index quintile for each survey respondent, from the poorest, to poor, medium, wealthy, and wealthiest quintile. In northern provinces (Hai Phong and Thai Nguyen), the majority of the caregivers were from the medium to the wealthiest quintiles, while in the center and the southern provinces (Quang Nam and Ca Mau), they were mainly from the poorest to the medium quintiles (*p* < 0.001). The MNP purchase trends by caregivers and by the wealth index quintiles are represented in Figure 5.

There was a positive correlation between the number of MNP sachets purchased and the wealth index (*r* = 0.434, *p* < 0.001).

## 4. Discussion

The results illustrate that the pilot project model of the locally manufactured MNP Bibomix, via the public health system, was successful in attaining the project goals. It was indeed the first time in the country that a MNP produced by the NIN was distributed for sale through the local health centers, and the MNP coverage results and financial sustainability are very promising.

The overall effective coverage of the project was 11.5% among all surveyed caregivers (and 27.3% among the ones who had visited the health center in the 30 days prior to the survey), which was higher than the initial projection of 8% effective coverage. In addition, 75.0% of the caregivers who had heard of Bibomix purchased it and gave it to their child, demonstrating a high conversion rate from product awareness to product trial. The MNP coverage in the province in the south (Ca Mau) did not reach the 8% target, with an effective coverage of only 5.1%. This was due mainly to the fact that fewer than 20% of caregivers in Ca Mau had ever heard about Bibomix, and the main reason was that they had difficulties accessing a health center. Indeed, only 18.2% of the caregivers surveyed had visited the health center within the last five months, and this could be explained because, unlike the other provinces, Ca Mau has an extensive network of canals from the Mekong Delta, which isolates the health centers from the village/commune and makes it more difficult to access for caregivers.

Therefore, while the health system was a reliable and trusted channel for the caregivers, one of the weaknesses and limitations of this pilot model was adapting for differentiated access to health centers. The MNP distribution intervention did not reach caregivers who did not have access to the local public health centers. For these specific groups (especially the ethnic minorities) who are “left behind” in the current public health system, a complementary delivery model to reach them is required.

The purchase trends also showed that caregivers in Northern provinces preferred buying a full box of Bibomix, while in the center and southern provinces they purchased mainly the intermediate packet and primarily single sachets respectively. Wealth index varies significantly by provinces, as the majority of caregivers were from the medium to the wealthiest quintiles in the northern provinces, while in the center and southern there were mainly from the poorest to the medium quintiles. The number of sachets bought by the caregivers was positively correlated with the wealth index, demonstrating that the innovation in the packaging size, *i.e.*, offering single sachets, packets of 10 sachets or boxes of 60 sachets, proved to be well suited to adapt to different purchasing powers. The different price points helped increase the product affordability and can be considered an important factor in driving trial of the MNP. As the coverage survey was conducted only five months after the start of the MNP sales at the health centers, repurchase trends by caregivers could not be assessed.

A majority of the health workers reported encountering difficulties when selling Bibomix to caregivers. These barriers were related mainly to the product Bibomix itself, where health workers reported caregiver concerns about the product usage/dosage, the lack of visible impact in the short term, and attributing side effects. Addressing these barriers would require further investment in awareness building and messaging (such as related long-term use of Bibomix to see health benefits) through continued counseling by the health workers. In addition, the distribution channel through commune health centers, while promising, was not yet “optimal” during the pilot, as there were reports of difficulties related to supply/logistics, such as budgeting for payment for supplies based on order rather than sales and transportation issues, which would require review and further training to better optimize the MNP supply and cash flow, and especially on forecasting and monitoring.

The strategy used in the pilot to strengthen the health system by increasing the capacity of health workers to counsel on IYCF and home fortification practices effectively was successful, and embed the distribution of MNPs in existing platforms. The BCI implementation showed that, through investment in training with supportive communication and education materials, IYCF and home fortification with MNPs messaging was effectively transferred to caregivers by interpersonal communication activities, such as counseling by the health workers. Indeed, the high compliance rate in the product use among the caregivers (59.0%) indicates that recommended behaviors were well communicated by the health staff, who was the primary source of credible information, to the caregivers. The MNP was correctly used in mashed/semi-solid foods even for children over two years old in most cases. This interesting result demonstrates that, while some caregivers complained about the MNP application not being adapted for children over two years old (as they no longer eat mashed or semi-solid food at that age), proper health staff training on product use demonstration was reflected in counseling to the caregivers that overcame established practices.

A limitation of the coverage survey was that, while the questionnaire was developed to have harmonized questions for countries implementing MNP programs globally, some relevant indicators that would have helped better understand caregivers’ behaviors/purchase trends/use of Bibomix in Vietnam were missing. For instance, data on ethnicity of the caregivers were missing, which would have allowed segmenting by ethnic minority groups. In addition, information about the frequency of visits to the health centers was not collected. Another limitation was that all the data were collected through face-to-face interviews with caregivers at the health centers, and not at the home. Caregivers of the selected children were invited to come at the CHC, which could have biased the responses given because they may have wanted to please the surveyors. Caregivers were asked to report consumption within one week, which is commonly used in dietary assessment, however may not be representative of habitual practice. The use of one-week recall methodology was chosen to avoid recall bias. In addition, as this survey was conducted in a programmatic setting, budget, and logistics constraints did not allow measurement and tracking of consumption per child, per week. Finally, for health worker routine monitoring, the main limitation was that the health staff did not always use the recording templates that were designed for the project, as they preferred using the recording form that they were already accustomed to. As a result, they could not have recorded the identity of the primary caregivers buying Bibomix, which would have been useful for following up the purchase and repurchase trends.

The findings of this pilot were very promising and subsequent to the pilot, Bibomix MNP was endorsed by the MOH (Decision 4994/QD-BYT on 27 November 2014) in its strategy for micronutrient deficiencies prevention. This policy change is the first key milestone to scaling up MNP distribution in the country using the model piloted through this project. It is also noteworthy that while the pilot ended at the end of December 2014, the counseling services, as well as MNP sales, are still running in the provinces, truly demonstrating political will and ultimately the sustainability of the model.

## 5. Conclusions

This pilot of home fortification of complementary food with MNPs reflected initiative and innovation on the part of the government of Vietnam to introduce an economically viable model for ensuring adequate micronutrient intakes by older infants and young children. The pilot tested a delivery model based entirely on the public health system. Bibomix for sale at health facilities with different packaging sizes and price points, which helped to adapt to each caregiver’s disposable income, was a key feature of the success of this strategy. While very promising, further assessment is required to: (1) improve the current model, with special focus on forecasting and distribution, and elaborate a national plan for scale up the delivery of the MNP across all provinces in the country; (2) expand the behavior change interventions to reach all potential caregivers with children under five years of age; and (3) design a complementary delivery model to extend the effective reach to vulnerable groups, including ethnic minorities, and those that are geographically marginalized, and therefore, have limited access to the health system. These are key for advocating for scaling-up home fortification of complementary foods with MNP, complementing the current interventions already implemented in the country that are aimed at improving the nutritional status of children under five years of age.

## Figures and Tables

**Table 1:** Micronutrient composition per 1 g sachet of MNP.

Micronutrient	Amount
Vitamin A	400.0 µg
Vitamin D	5.0 µg
Vitamin E	5.0 mg
Vitamin C	30.0 mg
Vitamin B1	0.5 mg
Vitamin B2	0.5 mg
Niacin	6.0 mg
Vitamin B6	0.5 mg
Vitamin B12	0.9 mg
Folic Acid	150.0 µg
Elemental Iron (as Pyrophosphate)	10.0 mg
Zinc	4.1 mg
Copper	0.56 mg
Selenium	17.0 µg
Iodine	90.0 µg

**Table 2:** Summary of monitoring and evaluation design, sampling size, and implementation timeframe.

	Routine Monitoring Data	Health Worker Monitoring Interviews	Health Workers in Depth Interviews	Caregivers Coverage Survey
Target	Health workers	Health workers	Health workers	Caregivers
Design	Monthly reporting by provinces to NIN	Face-to-face structured interviews	Semi-structured interviews	Cross-sectional survey Structured interviews
Key objective	Monitor MNP sales in all health centers involved in MNP supply and delivery to caregivers	Monitor execution of product delivery, storage, distribution, and availability of behavior change materials in compliance with the training guidelines	Identify MNP sales trends and any bottlenecks in distribution channel	Assess MNP coverage (effective coverage, message coverage, contact coverage, and partial coverage; see survey terminology) and product use and compliance among children 6–60 months of age
Sample size	All 337 health centers	64 health centers (19%), randomly selected	120 local health workers from province, district, and commune levels	962 caregivers with children from 6 to 60 months
Settings	Four provincial health centers, 20 district health centers, and 313 CHCs	Four provincial health centers, 20 district health centers, and 40 CHCs, randomly selected	Four provinces (Thai Nguyen, Hai Phong, Quang Nam, and Ca Mau), 8 districts, and 32 communes, randomly selected	
Implementation time	June 2014 to December 2014	Mid-November to early December 2014	End of November to end of December 2014, after six months of MNP distribution	

**Table 3:** Characteristics of the 962 surveyed caregivers.

Variable	Mean (SD) or %
Sex of child, % female	46.9%
Caregiver with children 6 to 23 months, %	43.7%
Caregiver with children 24 to 60 months, %	56.3%
Age of child, mean	28.4 ± 13.5 months
Child 6–23 months with continued breastfeeding, %	52.6%
Breastfed child 6–23 months with minimum acceptable diet, %	54%
Non-breastfed child 6–23 months with minimum acceptable diet, %	57%
Female caregiver, %	*n* = 956 mothers (99.4%)
Mean age of primary caregiver	29.36 ± 5.34 years old
Caregiver has at least finished primary school, %	99.1%
Caregiver’s occupation is farming, %	41.5%
